# Zinc Supplementation Stimulates Red Blood Cell Formation in Rats

**DOI:** 10.3390/ijms19092824

**Published:** 2018-09-18

**Authors:** Yen-Hua Chen, Hui-Lin Feng, Sen-Shyong Jeng

**Affiliations:** 1 Department of Basic Medicine, The Center of Translational Medicine, Xiamen Medical College, Xiamen 361023, China; yanhua09123@hotmail.com; 2 Department of Food Science, College of Life Sciences, National Taiwan Ocean University, Keelung 20224, Taiwan; fhl1430@gmail.com

**Keywords:** anemia, erythropoiesis, phenylhydrazine hydrochloride, rat, red blood cells, zinc, zinc signaling

## Abstract

In rats, mice, and humans, it is known that zinc deficiency may be related to anemia, and zinc supplementation influences hemoglobin production. Our previous studies indicate that in fish, zinc supplementation stimulates red blood cell (RBC) formation (erythropoiesis). However, it is not clear whether the mechanism of zinc-induced erythropoiesis stimulation in fish also occurs in rats. We induced anemia in rats using phenylhydrazine (PHZ) and injected either saline or ZnSO_4_ solution. We found that an appropriate amount of zinc stimulated erythropoiesis in the PHZ-induced anemic rats. The effects of ZnSO_4_ injection were dose-dependent. When the concentration of ZnSO_4_ was higher than 2.8 mg zinc/kg body weight, the RBC level of the anemic rats increased from 60 ± 7% to 88 ± 10% that of the normal rats in two days. Rat bone marrow cells with or without ZnCl_2_ supplementation were cultured in suspension in vitro. In the cell culture when the zinc concentration was at 0.3 mM, a 1.6-fold proliferation of nascent immature reticulocytes (new RBCs) was observed after one day. In the rat blood, zinc was combined with serum transferrin to induce erythropoiesis. The stimulation of RBC formation by zinc appears to be common among different animals.

## 1. Introduction

Zinc is an essential nutritional element for humans [1]. Zinc deficiency can significantly impact human health and clinical manifestations [2,3,4]. Mammalian RBCs are continuously replenished through erythropoiesis. A deficit in the total amount of RBCs is defined as anemia [5]. Zinc is considered as an essential factor for erythropoiesis in addition to iron, folate, and vitamin B_12_ [6]. Studies have indicated that anemia is significantly associated with zinc deficiency, especially in pregnant women [7,8,9,10,11,12,13,14] and preschool children [14,15,16,17,18,19]. To treat human zinc deficiency diseases, many zinc supplementation studies have been performed. The benefits and risks of zinc supplementation have also been reviewed [20,21,22,23]. Zinc in combination with iron supplementation was found to increase hemoglobin levels to a higher extent than iron alone in patients with iron-induced anemia [24,25,26]. Patients with chronic kidney disease (CKD) usually exhibit severe anemia complications because their kidneys cannot produce sufficient erythropoietin (EPO). Currently, recombinant human erythropoietin (rHuEPO) is used to treat renal anemia [27,28]. In addition to anemia, CKD patients usually have much lower zinc levels in their serum [29]. Zinc therapy has been used for CKD patients, and zinc supplementation in hemodialysis patients has been shown to increase hemoglobin levels [30,31,32,33,34]. These studies indicate that zinc supplementation influences hemoglobin production.

Using an experimental animal model, zinc-restricted rats, the uptake of zinc was shown to increase in rat bone marrow [35]. Furthermore, in zinc-deprived pregnant rats, erythrocyte production is suppressed [36]. Cells of erythroid lineage in the mice declined accompanied zinc-deficiency [37]. Additionally, in chronic zinc-deficient mice, the erythroid compartment was reduced [38]. In zinc-deficient rats, plasma EPO concentrations were shown to be markedly decreased, and RBC production was repressed [39,40,41]. These studies suggest that, in rats or mice, an appropriate amount of zinc is needed for erythropoiesis. Although select minerals influencing the hematopoietic process have been reviewed recently [42], no direct animal studies have shown that zinc induces RBC formation in rats.

There are several ways to produce experimental animal models of anemia to test the effects of zinc supplementation on new RBC production, e.g., phenylhydrazine hydrochloride (PHZ) treatment, bleeding of the animals [43]. PHZ causes the oxidative denaturation or hemolysis of RBCs. PHZ-induced anemia has been used as a model for the study of the hematological response of new agents [44,45]. In the present study, we generated PHZ-induced rats with anemia and injected saline or ZnSO_4_ solution to determine whether zinc supplementation could stimulate erythropoiesis in rats in vivo. In addition, rat bone marrow cells were cultured in suspension in vitro with or without ZnCl_2_ supplementation. Here, we present evidence that zinc stimulates RBC formation in rats in vivo and in vitro.

## 2. Results

### 2.1. Zinc-Stimulated Erythropoiesis in PHZ-Induced Anemic Rats

#### 2.1.1. Induction of Anemia in Rats by PHZ Treatment and the Effects of ZnSO_4_ on Their Hematology

**Figure 1:**
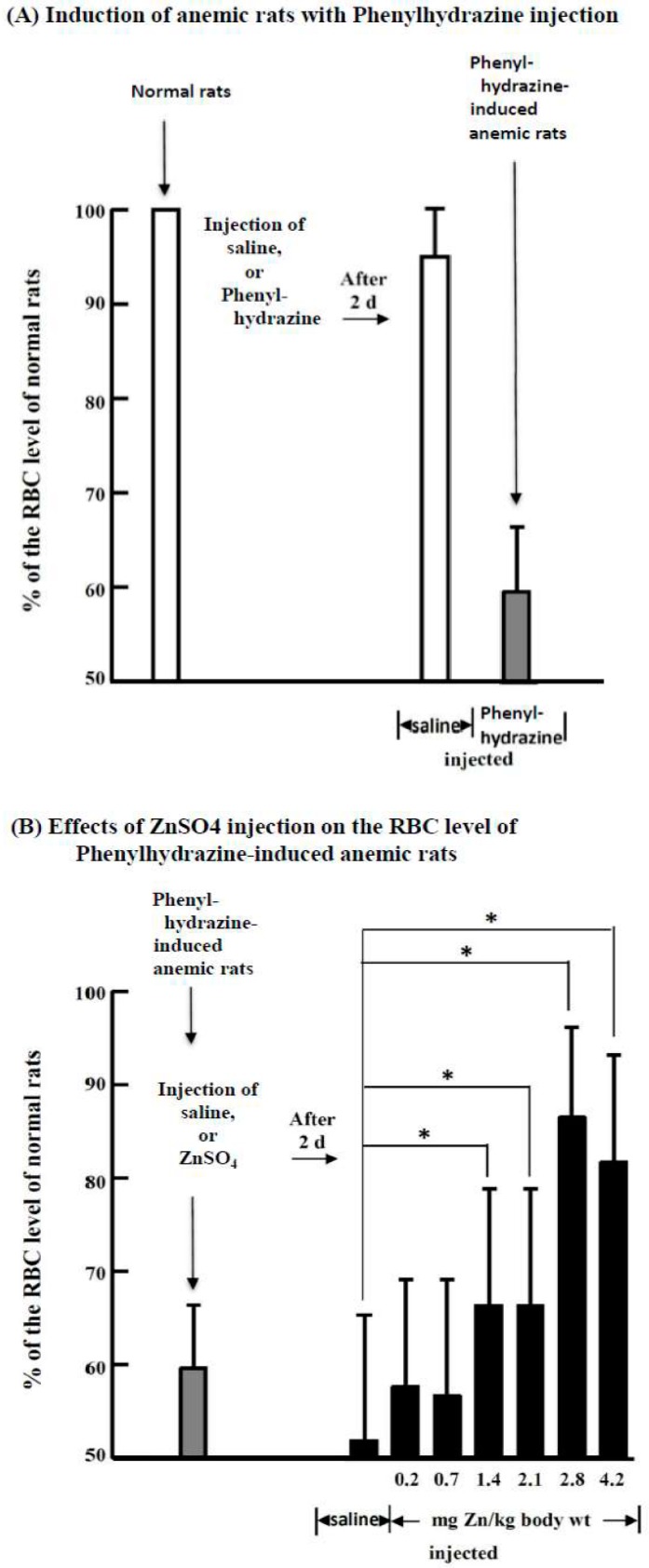
Induction of anemic rats by treatment with phenylhydrazine hydrochloride (PHZ) and the effects of ZnSO_4_ on their RBC levels. (**A**) Normal rats were injected with saline or a single PHZ (60 mg/kg body weight) solution at day 0; (**B**) After two days, the PHZ-induced anemic rats were further injected with saline or different concentrations of ZnSO_4_ solution. After another two days (at day 4), the rats were sacrificed, and RBC levels and other hematological indices were measured. *, significant difference between ZnSO_4_-injected and saline-injected anemic rats (*p* < 0.05, *n* = 6). This figure is representative of three different experiments.

Hematological studies on normal, saline-injected and ZnSO_4_-injected PHZ-induced anemic rats were performed (Table Sl). The most notable difference between saline- and ZnSO_4_-injected PHZ-induced anemic rats was the RBC counts. Figure 1 indicates that when the normal rats were injected with a single bolus of PHZ (60 mg/kg body weight), a rapid decrease in RBCs to 60 ± 7% that in normal rats was observed in two days (Figure 1A). When the PHZ-induced anemic rats were further injected with saline or different doses of ZnSO_4_ solution, after two days, the RBC level in saline-injected rats further dropped to 52 ± 14%; however, the ZnSO_4_-injected rats had significantly higher RBC proportions than the saline-injected rats when the zinc concentration was higher than 1.4 mg/kg body weight. When the injected zinc concentration was 2.8 mg/kg body weight, the RBC level recovered to 88 ± 10% of that in the normal rats (Figure 1B). As a control for the PHZ-induced anemic rats, normal rats were injected with 2.8 mg/kg body weight ZnSO_4_ solution, and after 2, 4, and 8 d, rat blood was examined. We found that the hematological data of the normal rats injected with ZnSO_4_ remained constant throughout the experiment (Table S2).

#### 2.1.2. Microscopic Observation of Blood Cells of Normal Rats, Saline-Injected Rats, and ZnSO_4_-Injected PHZ-Induced Anemic Rats

**Figure 2:**
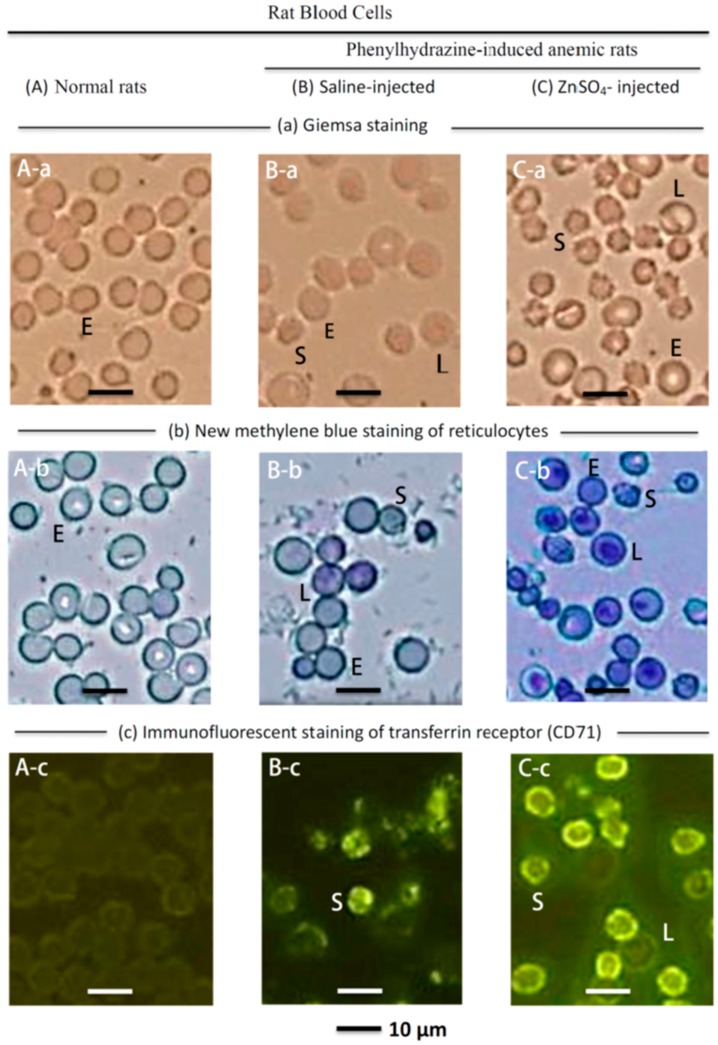
Microscopic observation of the blood cells of (**A**) normal rats; (**B**) saline-injected; and (**C**) ZnSO_4_-injected PHZ-induced anemic rats. (**A-a**) Giemsa staining indicated in normal rats virtually all the blood cells are mature erythrocytes (median cell diameter, 7.4 μm) (E). (**B-a**) Indicates that in the blood of the saline-injected PHZ induced anemic rats, many mature erythrocytes disappeared, but some small ‘star’ cells (median cell size 5.1 μm) (S), and some larger cells (median cell size 8.5 μm) (L) increased. (**C-a**) Indicates that in the blood of the 2.8 mg Zn/kg bd wt. ZnSO_4_-injected PHZ-induced anemic rats, more ~5 μm cells (S) proliferated. (**A-b**) New methylene blue staining indicated that in the mature erythrocytes, the cytoplasm is clear; however, in (B-b) and (**C-b**) the ~5 μm cells (S) and ~8.5 μm cells (L) have curved linear structures or dark blue dots in the cytoplasm with distinctive staining of the reticulocytes. (**A-c**) When the rat blood cells were immunofluorescently stained with CD71 antibody, mature erythrocytes (E) were not stained. However, (**B-c**) and (**C-c**) indicate that the ~5 μm cells (S) show strong immunofluorescence characteristics of immature reticulocytes, and the ~8.5 μm cells (L) showed faint immunofluorescence characteristics of mature reticulocytes.

Figure 2 shows the blood cells of normal rats and PHZ-induced anemic rats injected with saline or ZnSO_4_. The major blood cells in normal rats are mature erythrocytes (medium cell diameter, 7.4 μm) (E) (Figure 2A-a), and the major cells in the blood of ZnSO_4_-injected PHZ-induced anemic rats are ‘star’ cells (median cell size 5.1 μm) (S), and some larger cells (median cell size, 8.5μm) (L), besides the 7.4 μm mature erythrocytes (E) (Figure 2C-a). A comparison of Figure 2A-a and A-b with Figure 2B-a and B-b indicates that the saline-injected PHZ-induced anemic rats lost most of their mature erythrocytes (E) but exhibited an increase in ~5 μm cells (S) and some ~8.5 μm cells (L). The ZnSO_4_-injected anemic rats also lost most of their mature RBCs, but had substantially increased numbers of ~5 μm cells (S). Immature erythrocytes in the peripheral blood showed reticular, mesh-like structures when observed by microscopy with new methylene blue staining and are named reticulocytes [46]. The differentiation of erythroblasts to erythrocytes is accompanied by a marked change in membrane organization. During the differentiation into orthochromatic erythroblasts and reticulocytes, CD71 (transferrin receptor) is highly expressed [47]. Because CD71 is expressed on immature erythrocytes but not on mature erythrocytes, it is used as a marker of immature erythrocytes [48]. During the reticulocyte maturation process, the expression of CD71 antigen decreases; thus, the CD71 highly expressed reticulocytes are defined as immature reticulocytes, while the reticulocytes that faintly expressed CD71 are defined as mature reticulocytes [46]. The ~5 μm cells (S) in Figure 2C-a, C-b, and C-c were identified as immature reticulocytes because they showed a curved linear structure in the cytoplasm after new methylene blue staining (Figure 2C-b) and exhibited strong immunofluorescence after staining with a CD-71 antibody (Figure 2C-c). The ~8.5 μm cells (L) in Figure 2C-a, C-b, and C-c were identified as mature reticulocytes because they showed dark blue dots in the cytoplasm after new methylene blue staining (Figure 2C-b) and faint immunofluorescence after staining with a CD-71 antibody (Figure 2C-c).

#### 2.1.3. Measurement of Reticulocytes of Normal Rats, Saline-Injected PHZ-Induced Anemic Rats, and ZnSO_4_-Injected PHZ-Induced Anemic Rats with an Automatic Hematology Analyzer

To quantify the reticulocytes in the three experimental groups, the blood samples were evaluated using an automatic hematology analyzer. Table 1 indicates that the total reticulocyte level in normal rats was only 0.54 ± 0.12 × 10^6^ cells/mm^3^, while that in saline- and ZnSO_4_-injected PHZ-induced anemic rats was 1.28 ± 0.43 (2.4-fold) and 1.79 ± 0.42 × 10^6^ cells/mm^3^ (3.3-fold), respectively. The ZnSO_4_-injected rats had 1.4-fold (*p* < 0.05) higher proportion of total reticulocytes than the saline-injected rats. Among the different fractions of reticulocytes, the ZnSO_4_-injected rats had 1.3-fold (*p* < 0.05) higher HFR reticulocyte (immature reticulocytes) proportions than the saline-injected rats (1.08 ± 0.06 vs. 0.83 ± 0.04 × 10^6^ cells/mm^3^).

### 2.2. Biochemical Properties of the Blood of the ZnSO_4_-Injected PHZ-Induced Anemic Rats

#### 2.2.1. Change in the Zinc Level in the Blood of the ZnSO_4_-Injected PHZ-Induced Anemic Rats

The blood of the normal rats, saline-injected PHZ-induced anemic rats, and the 2.8 mg zinc/kg body weight-injected PHZ-induced anemic rats were collected, and the zinc levels in the plasma and blood cells were analyzed. Table 2 indicates that there was no significant difference in plasma levels among the three groups. However, the ZnSO_4_-injected PHZ-induced rats had 1.3-fold higher zinc level (105 ± 22 vs. 79 ± 11 μg Zn/10^6^ blood cell, *p* < 0.001) in the outer membrane, and 1.5-fold higher zinc level (200 ± 42 vs 134 ± 51 μg Zn/10^6^ blood cell, *p* < 0.001) in inside blood cells than the saline-injected rats, respectively.

#### 2.2.2. Isolation and Identification of Zinc-Binding Protein from the Blood Cells of the ZnSO_4_-Injected PHZ-Induced Anemic Rats

**Figure 3:**
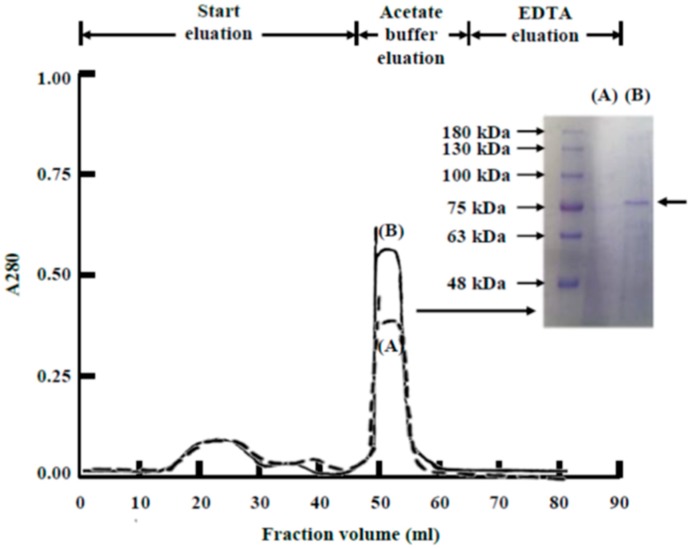
Isolation and identification of zinc-binding protein from the blood cells of ZnSO_4_-injected PHZ-induced anemic rats. Rat blood cells were collected from (**A**) normal rats; and (**B**) 2.8 mg Zn/kg body weight ZnSO_4_-injected PHZ-induced anemic rats. The cells collected were extracted using a detergent buffer, and the detergent extract was purified by Zn^2+^-IMAC. The acetate buffer eluates were pooled and analyzed by SDS-PAGE under reducing conditions, followed by staining with Coomassie blue to visualize the protein bands. The major difference between normal rats (**A**) and zinc-injected anemic rats (**B**) was the band with a molecular weight of 76 kDa in (**B**). The arrowhead denotes the position of the protein identified by nano-LC-MS/MS analysis as rat serum transferrin.

Because the blood cells in the 2.8 mg zinc/kg body weight ZnSO_4_-injected PHZ-induced anemic rats had substantially higher zinc levels than those in the normal rats, the zinc-binding substance in the blood cells of rats in the two groups was isolated and compared. Blood was collected from both groups and extracted with detergent. Then, the detergent extract was subjected to Zn^2+^-IMAC. The zinc-binding substance was eluted in acetate buffer (Figure 3). Coomassie blue-stained SDS-PAGE gels showed that a protein with an approximate molecular mass of 76-kDa was the major component of the acetate buffer eluent in the ZnSO_4_-injected PHZ-induced anemic rats. However, there was little or no protein in the normal rats. This 76-kDa protein was identified as rat serum transferrin using nano-LC-MS/MS. The sequence of rat serum transferrin has been reported, and the transferrin protein was reported to have a mass of 76,346 Da [49].

### 2.3. Zinc Salts Stimulated Erythropoiesis in Rat Bone Marrow Cells In Vitro

#### 2.3.1. Microscopic Observation of the Cultured Rat Bone Marrow Cells before and after ZnCl_2_ Supplementation

**Figure 4:**
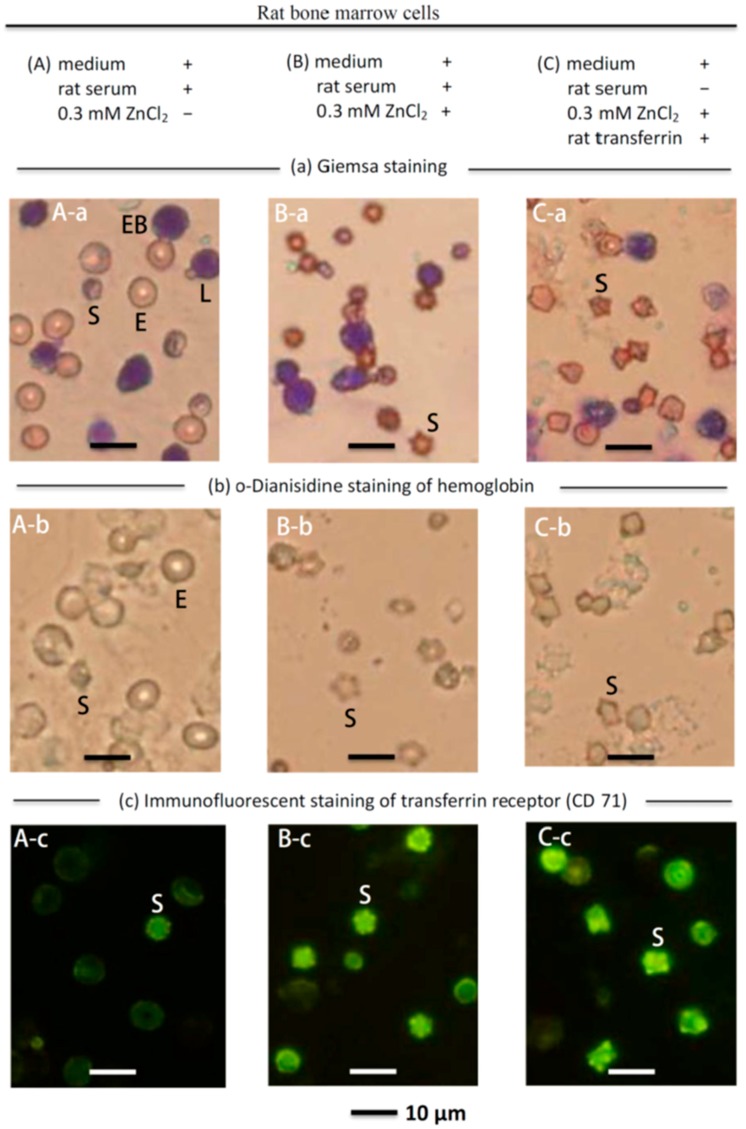
Microscopic observation of cultured rat bone marrow cells with ZnCl_2_ supplementation. (**A-a**) Giemsa staining of the rat bone marrow cells (with medium and rat serum) without supplementation of ZnCl_2_ suspension-cultured for one day. The major cells were mature erythrocytes with a median cell diameter of 7.4 μm (E), lymphocytes with a median cell diameter of 7.3 μm (L), erythroblasts (EB), and smaller ~5 μm cells (S); (**A-b**) o-Dianisidine staining (to stain erythroid cells) indicated that mature erythrocytes (E) and the ~5-μm cells (S) were stained; (**A-c**) In CD-71 immunofluorescent staining, only the ~5 μm cells (S) were stained; (**B-a**) Giemsa staining of the rat bone marrow cells (with medium and rat serum) supplemented with ZnCl_2_ and suspension-cultured for one day indicated that most of the original mature erythrocytes (E) disappeared but many ~5 μm cells (S) appeared, and the lymphocytes (L) remained unchanged; (**B-b**) o-Dianisidine staining indicated that the ~5 μm cells (S) appeared multilobular and stained positively for hemoglobin; (**B-c**) The ~5 μm cells (S) showed strong immunofluorescence when stained with the CD-71 antibody; (C-a) Giemsa staining of the rat bone marrow cells in serum-free medium supplemented with ZnCl_2_ and rat transferrin suspension-cultured for one day. The cell pattern in (**C-a**) was similar to that of (**B-a**), i.e., many ~5 μm cells (S) appeared, and most of the original mature erythrocytes (E) disappeared. (**C-b**) o-Dianisidine staining indicated that the ~5 μm cells (S) appeared to be multilobular and stained positively for hemoglobin like those in (**B-b**); (**C-c**) The ~5 μm cells (S) showed strong immunofluorescence when stained with a CD-71 antibody.

To determine whether zinc could stimulate erythropoiesis in rat bone marrow cells in vitro, rat bone marrow cells (with medium and rat serum) were cultured with or without ZnCl_2_ for one day, as shown in Figure 4A,B, respectively. The most notable change after the culture was supplemented with ZnCl_2_ was the appearance of many ‘star’ cells (cell size, ~5 μm) (S) and the disappearance of most of the mature erythrocytes (E) (Figure 4B-a). o-Dianisidine staining has been used to stain hemoglobin [50]. Figure 4B-b indicates that the ~5 μm cells (S) contained hemoglobin. The ~5 μm cells (S) also showed immunofluorescent staining of the transferrin receptor (CD-71) (Figure 4B-c). These cells were stained by o-Dianisidine and the CD-71antibody, and these newly proliferated ~5 μm cells (S) were identified as immature reticulocytes. In the previous section, it was found that the active substance in rat serum that combined with zinc was serum transferrin (Figure 3); therefore, in a serum-free medium, we cultured rat bone marrow cells with zinc and rat transferrin as shown in Figure 4C. Figure 4C-a shows that after being cultured for one day, many new ‘star’ cells (S) emerged. These ~5 μm cells (S) showed the presence of hemoglobin (Figure 4C-b) and the transferrin receptor CD-71 (Figure 4C-c). Overall, the pattern in Figure 4C is virtually identical to that in Figure 4B. These data verified that, in rat serum, transferrin was the active component combined with zinc to stimulate erythropoiesis.

#### 2.3.2. Cell Size and Number in Rat Bone Marrow Cell Suspension Culture before and after ZnCl_2_ Supplementation

**Figure 5:**
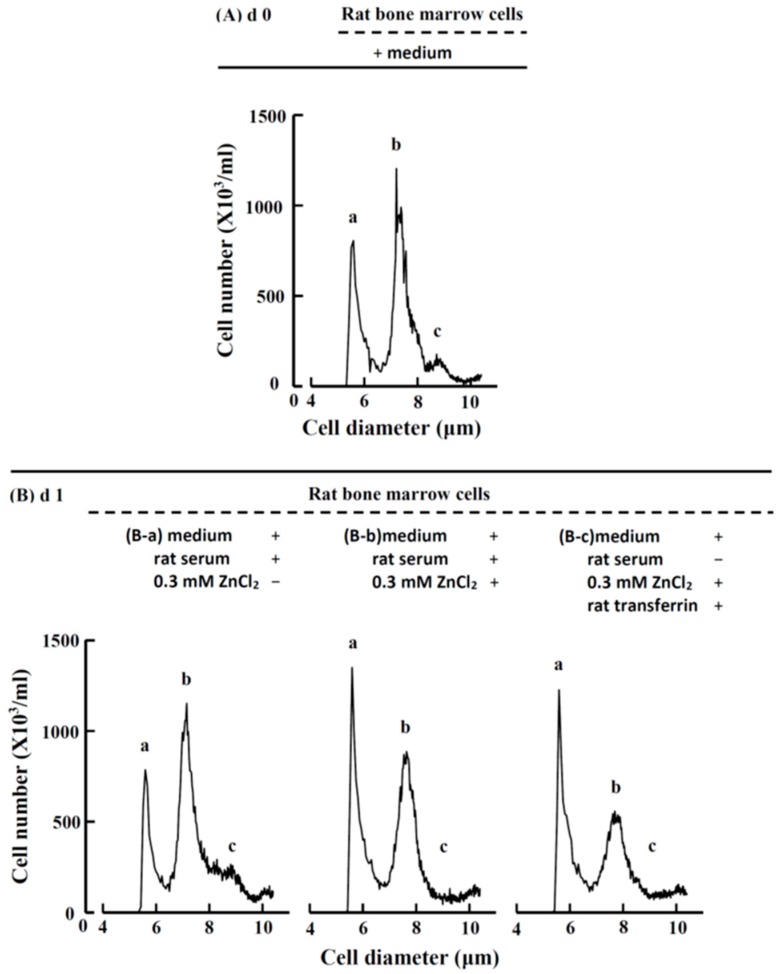
Cell diameter and number before and after zinc or rat transferrin supplementation of the rat bone marrow cell culture. (**A**) At day 0, the rat bone marrow cells (with medium) could be separated into three groups, namely, the ‘a’, ‘b’, and ‘c’ groups, which had peaks at 5.1, 7.4, and 8.5 μm, respectively; (**B-a**) After the cells were suspension-cultured with rat serum but without ZnCl_2_ for one day, the cell diameter and number did not change significantly; (**B-b**) When ZnCl_2_ was supplemented to the cells (with medium and rat serum), which were suspension-cultured for one day, the cell distribution changed greatly. The ‘a’ group cell number increased from 726 ± 42 to 1138 ± 188 × 10^3^/mL (*n* = 6) (1.6-fold, *p* < 0.01); however, the ‘b’ group cell number decreased from 913 ± 73 to 719 ± 94 × 10^3^/mL (*n* = 6) (0.8-fold, *p* < 0.05); (**B-c**) When ZnCl_2_ and rat transferrin were supplemented to the cells (with medium but without rat serum) for one day, the changes in cell distribution and cell number were quite similar to those in (**B-b**), i.e., the ‘a’ group cell number increased to 1058 ± 42 × 10^3^/mL (*n* = 6) (1.5-fold, *p* < 0.001) and the ‘b’ group cell number decreased to 563 ± 61 × 10^3^/mL (0.6-fold, *p* < 0.001) (*n* = 6). These figures are representative of six different experiments.

To quantify the change in cell size and number after the bone marrow cells were cultured with ZnCl_2_, the harvested cells were analyzed using an electron cell counter, as shown in Figure 5. The comparison of Figure 5A with the morphological observation in Figure 4A-a indicated that the ‘a’ group cells in Figure 5A were the ~5-μm cells (S); the ‘b’ group of cells consisted of mature erythrocytes (E) and lymphocytes (L), and the ‘c’ group of cells basically consisted of erythroblasts (EB). After the rat bone marrow cells (with medium and rat serum) were supplemented with 0.3 mM ZnCl_2_ and suspension-cultured for one day (Figure 5B-b), the cell distribution differed greatly from that of the cells without ZnCl_2_ (Figure 5B-a). The major change was that the ‘a’ group of cells (~5 μm cells) increased approximately 1.6-fold. When the rat bone marrow cells were suspension-cultured with ZnCl_2_ and rat transferrin in serum-free medium for one day, the cell distribution (Figure 5B-c) was virtually the same as that of Figure 5B-a. These results indicated that when rat bone marrow cells were suspension-cultured in vitro with 0.3 mM zinc for one day, the immature reticulocytes proliferated approximately 1.6-fold, and the blood zinc was combined with transferrin to stimulate erythropoiesis.

#### 2.3.3. Effects of ZnCl_2_ Levels in the Presence of Rat Serum on Cell Growth

**Figure 6:**
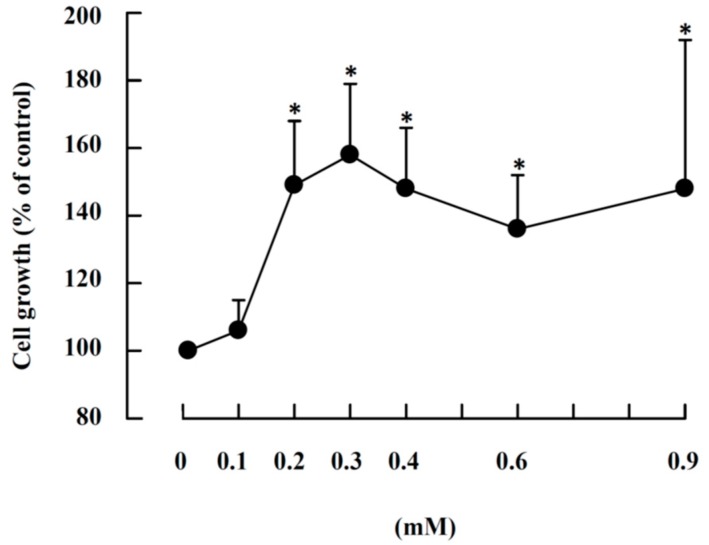
Effects of ZnCl_2_ concentrations on the growth of the 5.1 µm cells in the rat bone marrow cell culture. Rat bone marrow cells were suspension-cultured with 0.01 mM ZnCl_2_ (control) to 0.9 mM ZnCl_2_ in the presence of medium and 10% rat serum. After 24 h, the harvested cells were measured with a cell counter. Cell growth is expressed as the ratio of the number of 5.1-μm cells at the start of the culture compared with that of the control. The results are the mean ± SD from six independent experiments. *, significant difference between the ZnCl_2_ supplemented and control (*p* < 0.05, *n* = 6).

Because the growth of immature reticulocytes in the rat bone marrow cell culture could be expressed by the increase in the number of 5.1 μm cells (Figure 5); therefore, this parameter was used to measure cell growth. We studied the effects of ZnCl_2_ levels in the presence of rat serum on cell growth as shown in Figure 6. We observed significant activation of growth at 0.2 mM (149 ± 19% of the control, *n* = 6), and cell growth entered a plateau phase from 0.3 mM (158 ± 21% of the control, *n* = 6) to 0.9 mM. However, ZnCl_2_, or rat serum, or transferrin alone had little effect on cell proliferation; FeCl_3_ alone or FeCl_3_ + rat serum also had no effect on cell growth (Table S3).

#### 2.3.4. Effect of the Soluble EPO Receptor or a Rat EPO-Neutralizing Antibody on ZnCl_2_-Induced Erythropoiesis

**Figure 7:**
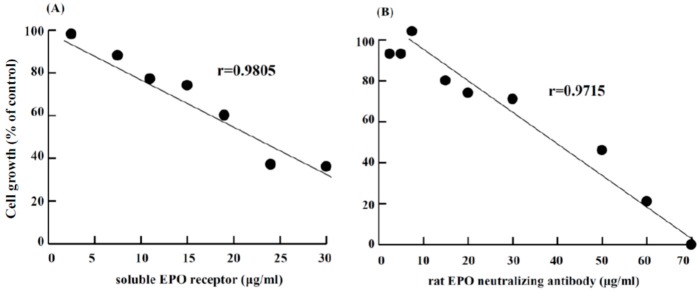
Effect of the soluble EPO receptor or rat EPO-neutralizing antibody on ZnCl_2_-induced erythropoiesis. Rat bone marrow cells were suspension-cultured with 0.3 mM ZnCl_2_ and supplemented with different concentrations of (**A**) soluble EPO receptor or (**B**) rat EPO-neutralizing antibody. After one day, the harvested cells were measured to determine the cell size and number. The cell growth is expressed as the ratio of the number of 5.1 μm cells at the start of the culture compared with that of the control.

To determine whether the stimulation of RBC formation by zinc in rat bone marrow cells occurred is related to EPO production, the cells were suspension-cultured with ZnCl_2_ and different levels of soluble EPO receptor or a rat EPO antibody. Figure 7 shows that erythropoiesis by ZnCl_2_ was inhibited by the soluble EPO receptor or rat EPO antibody in a dose-dependent manner. Approximately 60% or 100% of the cell growth was abrogated when the concentration of soluble EPO receptor was 30 μg/mL or the concentration of rat EPO-neutralizing antibody was 70 μg/mL, respectively.

## 3. Discussion

Previous studies indicated that zinc could induce erythropoiesis in fish [51,52,53,54], but the RBCs of fish maintain the nucleus, whereas mammalian RBCs differ fundamentally from those of fish and lack a nucleus. Thus, it was unclear whether the mechanism of zinc-induced erythropoiesis stimulation in fish also occurs in rats. However, in this report, we demonstrate that zinc also stimulates erythropoiesis in rats.

Based on hematological analyses (Figure 1), microscopic morphological observation (Figure 2), and quantification of reticulocytes with an automatic hematology analyzer (Table 1), it is clear that zinc stimulated RBC formation in PHZ-induced anemic rats when an appropriate amount of zinc was injected. The effects of ZnSO_4_ injection were dose-dependent; when the concentration was higher than 2.8 mg zinc/kg body weight, the RBCs of the anemic rats recovered from 60 ± 7% to 88 ± 10% of the normal level in two days. Quantitative analyses with an automatic hematology analyzer (Table 1) indicated that the 2.8 mg zinc/kg body weight ZnSO_4_-injected anemic rats had a 1.4- to 1.5-fold higher number of immature and mature reticulocytes than the saline-injected ones.

PHZ treatment is known to induce anemia in rats or mice [43,55]. Mice treated with 60 mg PHZ/kg body weight exhibit decreased hematocrit (Hct) levels from 47% (day 0) to 34% in four days and require approximately eight days for the Hct levels to return to basal levels [55]. In birds, a single injection of 12.5 μg PHZ/g body weight causes a rapid decrease in Hct and hemoglobin (Hb) within 24 h. Hct and Hb return to pre-injection levels within 5‒10 days of treatment [56]. In the present study, at least eight days were required for the RBCs of the PHZ-induced anemic rats to return to normal values if the anemic rats were injected only with saline (data not shown). Figure 1 indicates that on day 2, the PHZ-induced anemic rats had an RBC level of 60 ± 7% of the normal rats, and two days after a further injection of saline (at day 4) the RBC level further dropped to 52 ± 12%; therefore, regenerative erythropoiesis in rats did not occur within four days. Evidently, the recovery of RBC to the normal level after treatment with ZnSO_4_ in the present study is derived from stimulation by zinc, but not from regenerative erythropoiesis in rats at this time point.

It has been reported that the intraperitoneal injection of LD_50_ of zinc salts in rats is 28–73 mg/kg [5,57]. In the present study, the effective dose for the anemic rats to recover their RBCs to 88 ± 10% of the normal level was 2.8 mg/kg. Therefore, the zinc level needed to relieve anemia in rats was substantially lower than the reported LD_50_.

The present report found that zinc stimulated erythropoiesis not only in rats in vivo but also in rat bone marrow cells in vitro. When the zinc concentration in cell culture was higher than 0.2 mM, erythropoiesis was activated; when the zinc concentration was 0.3 mM, a 1.6-fold proliferation of nascent immature reticulocytes was observed in one day (Figure 4 and Figure 5). The in vivo experiment indicated that in ZnSO_4_-injected PHZ-induced anemic rats, increased zinc was bound by transferrin in blood (Figure 3). In vitro, when rat bone marrow cells were cultured with zinc and transferrin in a serum-free medium, RBCs proliferated similar to RBCs grown in medium supplemented with zinc + rat serum (Figure 4 and Figure 5). These results indicated that, in rat serum, transferrin was the active compound that interacted with zinc to stimulate erythropoiesis. Recently, it was reported that “zinc signal” is essential for physiology [58]. We reported that zinc is not only a signal for common carp to stimulate erythropoiesis, but also for many other fish [54]. In the present report, we found that this kind of mechanism also exists in rats. The stimulation of RBC formation by zinc signal appears to be common among different animals. However, the zinc concentration for maximum activation of RBCs in vitro seems differs in different animal species, e.g., in rats, it is 0.3 mM; for common carp, also 0.3 mM; but 0.6, 0.2, 0.3, and 1.2 mM for crucian carp, grass carp, silver carp, and tilapia, respectively [54]. Although the newly proliferated RBC cells of rats (Figure 4) and fish [54] all showed strong immunofluorescence when stained with the CD-71 antibody; the new reticulocytes of the rat blood have a ‘star’ shape (Figure 2) and have no nucleus, while the newly proliferated RBCs in the fish blood showed a round shape and contain a nucleus [54].

As mentioned above, when normal rats were injected with 2.8 mg/kg body weight ZnSO_4_, there was no change in the RBC count or Hct level after 2, 4, and 8 d (Table S2). Only when the rats were under anemic conditions did the injection of ZnSO_4_ induce erythropoiesis. It is possible that rats have a sophisticated regulatory system that is only active under anemic conditions, which causes the zinc‒transferrin complex in blood to be carried to the bone marrow, where erythropoiesis occurs.

Zinc supplementation in chronic kidney disease patients undergoing hemodialysis has been shown to increase hemoglobin levels [30,31,32,33,34]. These studies indicate that zinc supplementation influences hemoglobin production. Zinc supplementation in elderly men significantly increased Hct levels, RBC proportions, and testosterone levels. Therefore, it is suggested that the increase of RBCs might occur via androgen metabolism [59]. Zinc supplementation in pregnant women with anemia resulted in an increase in insulin-like growth factor-1 (IGF-1), which was correlated with an increase in Hb levels and RBCs. These results suggest that IGF-1 has a role in increasing RBC level [8,60]. Sato et al. [61] studied the role of EPO receptor (EPOR) in human erythropoiesis by neutralization of EPO using an anti-EPO mAb and other methods, and they found that erythroid cells themselves produced EPO and that they stimulated their own erythroid differentiation in an autocrine manner. Our preliminary experiment (Figure 7) shows that in vitro erythropoiesis by ZnCl_2_ in rat bone marrow cells was inhibited by the soluble EPO receptor or rat EPO antibody. Furthermore, we are not able to detect EPO immunoactivity in the cultured medium. Therefore, we suspected that the zinc-induced erythropoiesis in our study may be related to autocrine EPO. Reports have indicated that erythroid progenitors differentiate and mature in response to autocrine EPO in mice and humans [62,63,64]. It is not clear now whether the mechanism of zinc-induced erythropoiesis in our experiment is due to autocrine EPO or other factors. Although zinc is considered an important factor in erythroid differentiation [6], the detailed mechanism involved in zinc-induced erythropoiesis requires further investigation.

## 4. Materials and Methods

### 4.1. Animals

Adult male Sprague‒Dawley rats weighing 210 ± 20 g were used in all experiments. The rats were kept in the in-house animal facility, maintained at 22 ± 3 °C and 55% ± 5% relative humidity with a 12 h light‒dark cycle. The rats were maintained under laboratory environmental conditions with ad libitum access to tap water and food. Animal use was reviewed and approved by the Institutional Animal Care and Use Committee at National Taiwan Ocean University (No. 104036, 14 December 2015).

### 4.2. PHZ-Induced Anemic Rats and ZnSO_4_ Injections after Anemia Was Induced

Normal rats were injected with sterilized normal saline (0.9% NaCl) or a single PHZ (Sigma Chemical Co., St. Louis, MO, USA) in sterilized normal saline by the intraperitoneal route at 60 mg/kg body weight. After two days, a total of 42 PHZ-induced anemic rats were divided into seven groups: the control groups receiving saline solution intraperitoneally; and the experimental groups receiving 0.2, 0.7, 1.4, 2.1, 2.8, or 4.2 mg zinc/kg of ZnSO_4_ solution (*n* = 6, for each time point). After another two days (at day 4), the rats were sacrificed, and RBC levels and other hematological metrics were measured. Three independent experiments were performed.

### 4.3. Blood Sampling and Analysis

At each sacrifice time point, blood samples were immediately drawn by cardiac puncture of each rat. The hematological parameter was measured by an automatic hematological assay analyzer (Cell Excell 500; Danam Electronics, Dallas, TX, USA).

### 4.4. Measurement of Reticulocytes in Rat Blood

Reticulocytes were measured with ProCyteDx Hematology Analyzer (IDEXX Laboratories, Inc. Westbrook, ME, USA).

### 4.5. Microscopic Observation of Rat Blood Cells by Giemsa, New Methylene Blue, and o-Dianisidine Staining

Blood smears of normal, saline-injected, and ZnSO_4_-injected PHZ-induced anemic rats were prepared immediately after blood sample collection and directly fixed on a slide, stained with Giemsa [65] new methylene blue [66] or o-Dianisidine [50] and subjected to microscopic observation. The images were observed by an inverted microscope (Olympus IX71, Olympus Optical Co, LTD, Tokyo, Japan) and captured via an Olympus-DP70-Digital-microscope-Camera (Olympus Optical Co, LTD, Tokyo, Japan). The images were then processing by DP Controller/Manager software (Olympus Optical Co, LTD, Tokyo, Japan).

### 4.6. Isolation and Identification of the Active Substance in Rat Blood that Stimulates the Proliferation of RBC Cells in Rats

Preparation of rat blood from normal rats and 2.8 mg Zn/kg body weight ZnSO_4_-injected PHZ-induced anemic rats, detergent extraction of zinc-binding proteins from the blood cells, immobilized metal affinity chromatography (IMAC) purification of zinc-binding proteins, electrophoresis, and nano-LC-MS/MS were performed as reported previously [52].

### 4.7. Determination of Zinc Concentration in Different Blood Fractions

After whole blood was withdrawn by cardiac puncture with heparinized syringes, the blood was used to determine the zinc concentration in different blood fractions. Plasma was obtained by centrifuging 0.4 mL whole blood at 2000× *g* for 10 min. The precipitate was added to 3 mL 1.5 mM 1,10-phenanthroline in isotonic NaCl solution. The suspension was shaken for 30 min and centrifuged at 1500× *g* for 10 min. The supernatant was then removed, and the zinc concentration of this solution (which represented the zinc released from the outer plasma membranes of the blood cells) was measured [67]. The precipitate was added to 2 mL 6 mM EDTA in 50 mM Tris-HCl buffer, shaken for 15 min and centrifuged at 10,000× *g* for 10 min. The zinc concentration in this supernatant represents the zinc inside the blood cells. Zinc concentration was determined by atomic absorption spectrophotometry (Z-8100, Hitachi, Tokyo, Japan).

### 4.8. Suspension Culture of Rat Bone Marrow Cells with or without ZnCl_2_ Supplementation

Aliquots of 0.15 mL of bone marrow cell suspension (approximately 60 × 10^6^ cells/mL) were seeded in 24-well culture plates. An equal volume (0.15 mL) of DMEM/F12 medium with 20% rat serum with or without ZnCl_2_ supplementation was added. The cultures were incubated at 37 °C in an atmosphere of 5% CO_2_. At 0, 1, 2, and 3 d, the cells from each well of each group were harvested. The number and size of the cells from the harvested cell suspension were then determined with an electronic cell counter (Z2, Beckman Coulter, Hialeah, FL, USA). Eleven independent experiments were performed.

### 4.9. Suspension Culture of Rat Bone Marrow Cells in Rat Serum-Free Medium with or without Transferrin or ZnCl_2_ Supplementation

Aliquots of 0.15 mL of rat bone marrow cell suspension (approximately 60 × 10^6^ cells/mL) were seeded in a 24-well culture plate. An equal volume (0.15 mL) of DMEM/F12 medium with or without 0.6 mM ZnCl_2_ and with or without rat transferrin (0.2 μg/mL) (Sigma Chemical Co., St. Louis, MO, USA) was added. The cells were cultured as described above. At one day, the cells from each well of each group were harvested. The number and size of the cells from the harvested cell suspension were then determined. Six independent experiments were performed.

### 4.10. Microscopic Observation of Rat Bone Marrow Cells with Giemsa Staining

The harvested rat bone marrow cells were stained using the Giemsa method for light microscopic examination.

### 4.11. Fluorescence Staining of CD71 (Transferrin Receptor) in Rat Blood and Bone Marrow Cells

Slides of fixed rat blood cells or bone marrow cells were blocked with blocking buffer (0.2% BSA and 1% normal goat serum in PBS) for 1 h at room temperature and incubated at 4 °C overnight with a CD71 antibody (mouse anti-human transferrin receptor, clone H 68.4, 1:1000 dilution; Invitrogen, Camarillo, CA, USA). The slides were then incubated with a fluorescein isothiocyanate-conjugated anti-mouse IgG secondary antibody (Sigma Aldrich Chemie Gmbh, Schnelldorf, Germany). Negative controls were performed by incubating the cells with normal rat serum instead of a primary antibody. The cells were examined by fluorescence microscopy. No immune-reactivity in the rat blood or bone marrow cells was found in the negative controls (data not shown).

### 4.12. Effects of ZnCl_2_, FeCl_3_, and/or Transferrin on the Growth of RBC from the Rat Bone Marrow Cells

Aliquots of 0.15 mL of rat bone marrow cell suspension (60 × 10^6^ cells/mL) were added to equal volumes of DMEM/F12 medium with ZnCl_2_ (0 to 1.8 mM), with or without 20% rat serum, and with or without rat transferrin (0.2 μg/mL) (Sigma Chemical Co.), or FeCl_3_ (0.2 or 0.6 mM). The cells were cultured as described above. At days 0 and 1, the cells were harvested, and the number and size of the cells from the harvested cell suspension were determined. Cell growth was expressed as the ratio of the number of 5.1 μm cells relative to those of the control. Six independent experiments were performed.

### 4.13. Effects of EPO Antibody or Soluble EPO Receptor on Erythropoiesis

Aliquots of 0.15 mL of bone marrow cell suspension (60 × 10^6^ cells/mL) were added to equal volumes of DMEM/F12 medium with 0.6 mM ZnCl_2_, 20% rat serum, and different concentrations of soluble EPO receptor (0, 2.5, 7.5, 11, 15, 19, 24, and 30 μg/mL) (307-ER: R&D Systems, Minneapolis, MN, USA) or rat EPO antibody (0, 2.5, 5.0, 7.5, 15, 20, 30, 50, 60, and 70 μg/mL) (AF959: R&D Systems, Minneapolis, MN, USA). The cells were cultured as described above. At days 0 and 1, the cells were harvested, and cell size and number were determined. Cell growth was expressed as the ratio of the number of 5.1 μm cells relative to those of the control.

### 4.14. Data Analysis

The data are expressed as the mean ± standard deviation (SD). The significance of the experimental results was calculated using one-way analysis of variance, followed by the least significant difference post hoc test, using SPSS 10.0 (SPSS Inc., Chicago, IL, USA).

## Figures and Tables

**Table 1:** Measurements of reticulocytes in normal, saline-injected and ZnSO_4_-injected PHZ-induced rats with an automatic hematology analyzer.

RBC Count (10^6^ Cell/mm^3^)					
Total RBC	Mature Erythrocytes	Reticulocytes			
		Total	Fraction *		
			LFR	MFR	HFR
(A) Normal rats (*n* = 6) ^†^					
6.91 ± 0.91 ^‡^^,^^a^	6.37 ± 0.12 ^a^	0.54 ± 0.12 ^a^	0.22 ± 0.03 ^a^	0.14 ± 0.04 ^a^	0.18 ± 0.08 ^a^
(B) Saline-injected PHZ-induced anemic rats (*n* = 6)					
3.58 ± 0.95 ^b^	2.30 ± 0.43 ^b^	1.28 ± 0.43 ^b^	0.28 ± 0.03 ^b^	0.17 ± 0.01 ^a^	0.83 ± 0.04 ^b^
(C) 2.8 mg Zn/kg bd wt ZnSO_4_-injected PHZ-induced anemic rats (*n* = 6)					
6.08 ± 0.69 ^c^	4.29 ± 0.42 ^c^	1.79 ± 0.42 ^c^	0.43 ± 0.05 ^c^	0.29 ± 0.04 ^b^	1.08 ± 0.06 ^c^

* LFR, low-fluorescence reticulocytes; MFR, medium-fluorescence reticulocytes; HFR, high-fluorescence reticulocytes. ^†^ Number of rats; ^‡^ Mean ± SD; a, b, c: significant differences (*p* < 0.05) among the (A), (B), and (C) groups.

**Table 2:** Change of Zn level in the blood of the ZnSO_4_-injected PHZ-induced anemic rats.

	Plasma Zn (μg Zn/mL Plasma)	Blood Cell Zn (μg Zn/106 Bloodcells) *	
		Outer Plasma Membrane	Inside Blood Cells
(A) Normal rats (*n* = 6) ^†^	2.16 ± 0.48 ^‡^	48 ± 4 ^a^	80 ± 14 ^a^
(B) Saline-injected PHZ-induced anemic rats (*n* = 6)	2.13 ± 0.18 ^c^	79 ± 11 ^b^	134 ± 51 ^b^
(C) 2.8 mg Zn/kg bdwt ZnSO4-injected PHZ-induced anemic rats (*n* = 6)	2.00 ± 0.10 ^c^	105 ± 22 ^c^	200 ± 42 ^c^

* See Materials and Methods for fractionation of blood cells; ^†^ Number of rats; ^‡^ Mean ± SD; a, b, c: significant differences (*p* < 0.001) among the (A), (B), and (C) groups.

## Supplementary Materials

The following are available online at http://www.mdpi.com/1422-0067/19/9/2824/s1.

## Abbreviations

EPO	Erythropoietin
IGF-1	Insulin-like growth factor-1
IMAC	Immobilized metal affinity chromatography
PHZ	Phenylhydrazine
RBC	Red blood cell
