# Supplementary material for: Zinc Supplementation Stimulates Red Blood Cell Formation in Rats
Source: Int J Mol Sci. 2018 Sep 18;19(9):2824. doi: 10.3390/ijms19092824 (PMC6165144; doi:10.3390/ijms19092824)
Supplement: Supplementary file 1 [file ijms-19-02824-s001.pdf]

Table S1. Hematology of normal rats, PHZ-induced anemic rats, and saline- or $ZnSO_4$-injected PHZ-induced anemic rats.

Rat group	RBC (10^6cells/mm^3)	HCT (%)	Hb (g/dl)	MCV (μm^3)	MCH (pg/cell)	MCHC (g/dl)
1. Normal rats	6.91 ± 0.76	43.7 ± 5.7	15.6 ± 3.9	63 ± 7	23 ± 7	36 ± 7
2. PHZ-induced anemic rats	4.13 ± 0.49	23.6 ± 3.9	15.2 ± 0.9	71 ± 2	37 ± 2	52 ± 4
3. Saline-injected PHZ-induced anemic rats	3.58 ± 0.95	34.4 ± 3.9	14.7 ± 3.1	107 ± 37	41 ± 5	42 ± 12
4. $ZnSO_4$-injected PHZ-induced anemic rats						
0.2 mg Zn/kg bw injected	3.89 ± 0.13	36.4 ± 2.0	15.7 ± 0.4	105 ± 4	39 ± 2	42 ± 11
0.7 mg Zn/kg bw injected	3.91 ± 0.94	31.3 ± 4.7	15.3 ± 0.4	82 ± 10	41 ± 9	50 ± 6
1.4 mg Zn/kg bw injected	4.66 ± 0.16*	34.1 ± 4.7	14.5 ± 0.5	77 ± 3	31 ± 1*	40 ± 2
2.1 mg Zn/kg bw injected	4.64 ± 0.87*	36.7 ± 9.0	15.2 ± 2.9	80 ± 16	34 ± 8	43 ± 9
2.8 mg Zn/kg bw injected	6.08 ± 0.69*	35.2 ± 0.6	14.1 ± 0.8	59 ± 6*	23 ± 1*	40 ± 2
4.2 mg Zn/kg bw injected	5.72 ± 0.86*	36.5 ± 1.9	15.0 ± 0.2	65 ± 6*	27 ± 4*	41 ± 3

1. n = 6.
2. Two days after the normal rats were injected with a single PHZ (60 mg/kg body weight) solution (n = 6).
3. Two days after the PHZ-induced anemic rats were injected with a saline solution (n = 6).
4. Two days after the PHZ-induced anemic rats were injected with different concentration of ZnSO4 solution (n = 6 for each subgroup).

RBC: total red blood cell count, HCT: hematocrit, Hb: hemoglobin, MCV: mean corpuscular hemoglobin concentration (HCT/HCT×10), MCH: mean corpuscular hemoglobin (Hb/RBC×100), MCHC: mean corpuscular hemoglobin concentration (Hb/HCT×100) .

The data are expressed as the mean ± SD.

This table is representative of three different experiments.

*, significant difference between $ZnSO_4$-injected and saline-injected anemic rats ($p < 0.05$, n = 6).

Table S2. Hematology of the normal rats and the saline- or ZnSO4-injected normal rats.

Rat group	RBC (10^6cells/mm^3)	HCT (%)	Hb (g/dl)	MCV (μm^3)	MCH (pg/cell)	MCHC (g/dl)
1. Normal rats	7.00 ± 0.63	45.1 ± 2.4	16.8 ± 0.2	65 ± 2	24 ± 2	37 ± 2
2. Saline-injected	6.67 ± 0.50	40.2 ± 4.5	18.1 ± 1.0	61 ± 8	27 ± 3	46 ± 8
3. 2.8 mg Zn/kg bw $ZnSO_4$-injected normal rats						
After 2 days	7.19 ± 1.04	45.4 ± 6.7	17.7 ± 1.1	63 ± 3	25 ± 2	40 ± 5
4 days	7.00 ± 0.53	42.4 ± 4.9	17.0 ± 1.0	60 ± 4	24 ± 2	41 ± 6
8 days	6.67 ± 0.51	41.4 ± 2.2	17.1 ± 0.4	62 ± 3	26 ± 2	41 ± 2

1. n = 6.
2. Two days after the normal rats were injected with a saline solution (n = 6)
3. Two, 4, and 8 d after the normal rats were injected with 2.8 mg Zn/kg body weight of $ZnSO_4$ solution (n = 6 for each subgroup).

RBC: total red blood cell count, HCT: hematocrit, Hb: hemoglobin, MCV: mean corpuscular hemoglobin concentration (HCT/HCT×10), MCH: mean corpuscular hemoglobin (Hb/RBC×100), MCHC: mean corpuscular hemoglobin concentration (Hb/HCT×100).

The data are expressed as the mean ± SD.

Table S3. Suspension cultures of rat bone marrow cells with different supplementation. Cultured cells were harvested after 24 h and measured with a cell counter. Cell growth is expressed as the ratio of the number of 5.1-μg cells at the start of the culture compared with that of the control.

Supplementation	Cell growth	
	0 d	1 d
1. Medium only	1.00	1.22±0.10
2. a. medium+0.3 mM $ZnCl_2$	1.00	1.15±0.04
b. medium+10% rat serum	1.00	1.13±0.19
c. medium+0.1 μg/mL rat transferrin	1.00	1.16±0.05
3. a. medium+0.3 mM $ZnCl_2$ +10% rat serum	1.00	1.56±0.26***
b. medium+0.3 mM $ZnCl_2$ +0.1 μg/mL rat transferrin	1.00	1.46±0.06***
4. a. medium+0.1 mM $FeCl_3$	1.00	0.99±0.31
b. medium+0.3 mM $FeCl_3$	1.00	0.98±0.21
c. medium+0.3 mM $FeCl_3$ +10% rat serum	1.00	1.00±0.18
d. medium+0.3 mM $FeCl_3$ +0.1 μg/mL rat transferrin	1.00	0.96±0.17

Results are the mean ± SD from six independent experiments.
***; Significant difference ($P < 0.001$) between cell growth at 0 and 1 day.
